# Investigation and identification of functional post-translational modification sites associated with drug binding and protein-protein interactions

**DOI:** 10.1186/s12918-017-0506-1

**Published:** 2017-12-21

**Authors:** Min-Gang Su, Julia Tzu-Ya Weng, Justin Bo-Kai Hsu, Kai-Yao Huang, Yu-Hsiang Chi, Tzong-Yi Lee

**Affiliations:** 1 Department of Computer Science and Engineering, Yuan Ze University, Taoyuan, 320 Taiwan; 2 Department of Medical Research, Taipei Medical University Hospital, Taipei, 110 Taiwan; 3 Department of Medical Research, Hsinchu Mackay Memorial Hospital, Hsinchu City, 300 Taiwan; 4 Innovation Center for Big Data and Digital Convergence, Yuan Ze University, Taoyuan, 320 Taiwan

## Abstract

**Background:**

Protein post-translational modification (PTM) plays an essential role in various cellular processes that modulates the physical and chemical properties, folding, conformation, stability and activity of proteins, thereby modifying the functions of proteins. The improved throughput of mass spectrometry (MS) or MS/MS technology has not only brought about a surge in proteome-scale studies, but also contributed to a fruitful list of identified PTMs. However, with the increase in the number of identified PTMs, perhaps the more crucial question is what kind of biological mechanisms these PTMs are involved in. This is particularly important in light of the fact that most protein-based pharmaceuticals deliver their therapeutic effects through some form of PTM. Yet, our understanding is still limited with respect to the local effects and frequency of PTM sites near pharmaceutical binding sites and the interfaces of protein-protein interaction (PPI). Understanding PTM’s function is critical to our ability to manipulate the biological mechanisms of protein.

**Results:**

In this study, to understand the regulation of protein functions by PTMs, we mapped 25,835 PTM sites to proteins with available three-dimensional (3D) structural information in the Protein Data Bank (PDB), including 1785 modified PTM sites on the 3D structure. Based on the acquired structural PTM sites, we proposed to use five properties for the structural characterization of PTM substrate sites: the spatial composition of amino acids, residues and side-chain orientations surrounding the PTM substrate sites, as well as the secondary structure, division of acidity and alkaline residues, and solvent-accessible surface area. We further mapped the structural PTM sites to the structures of drug binding and PPI sites, identifying a total of 1917 PTM sites that may affect PPI and 3951 PTM sites associated with drug-target binding. An integrated analytical platform (CruxPTM), with a variety of methods and online molecular docking tools for exploring the structural characteristics of PTMs, is presented. In addition, all tertiary structures of PTM sites on proteins can be visualized using the JSmol program.

**Conclusion:**

Resolving the function of PTM sites is important for understanding the role that proteins play in biological mechanisms. Our work attempted to delineate the structural correlation between PTM sites and PPI or drug-target binding. CurxPTM could help scientists narrow the scope of their PTM research and enhance the efficiency of PTM identification in the face of big proteome data. CruxPTM is now available at http://csb.cse.yzu.edu.tw/CruxPTM/.

**Electronic supplementary material:**

The online version of this article (10.1186/s12918-017-0506-1) contains supplementary material, which is available to authorized users.

## Background

Proteins are the major functional molecules in living cells, playing essential roles in various cellular processes such as catalysis, transport, and structural integrity. Although the human genome is estimated to harbor approximately 25,000 genes [1], alternative splicing of transcripts and post-translational modifications (PTMs) of proteins result in millions of proteins with diverse functions [2, 3]. PTMs regulate a protein’s function, level, and activity through the covalent attachment of small chemical molecules to certain amino acid residues, allowing proteins to respond to developmental signals or environmental stimuli [4, 5]. A protein’s structure can also be altered by these site-specific chemical modifications, leading to changes in stability, localization, and associations with other interacting molecules [6].

Recent advancement in high-throughput mass spectrometry (MS)-based proteomics technology has facilitated the identification of more than 200 different PTMs [7]. Many databases [6, 8–14] and tools [15–31] have been proposed for characterizing and identifying the substrate sites of a specific PTM type. Regarding the structural investigation of PTM sites, Zanzoni et al. have built a database of three-dimensional structures of protein phosphorylation sites (Phospho3D) in 2007 [15]. As an update to Phospho3D [16] published in 2011, Su et al. made a successful attempt at uncovering kinase-associated phosphorylation sites on the three-dimensional structure of proteins, by incorporating information such as spatial amino acid composition and substrate sequence motifs [17]. In 2014, Su et al. developed a new resource (topPTM) that considers transmembrane topology on 3D structures for the identification of functional PTM sites on membrane proteins [18]. Additionally, Craveur et al. designed a database (PTM-SD) for accumulating structurally resolved PTMs in proteins [19]. Although several databases were dedicated to characterizing the structures of PTM sites on protein tertiary structures, there exists no resource currently for providing an integrative platform to explore the PTM sites associated with drug binding and protein-protein interaction. According to the PTM data presented in UnitProtKB [20] and dbPTM [6], Table 1 shows the data statistics of PTM sites involved in protein-protein interaction (PPI), including drug binding. It appears that at least 65% (13,080/20,186) of known human proteins are regulated by PTM. More than 70% of human proteins can undergo PTM and may interact with other proteins. At the same time, over 70% of human proteins represent drug targets and can be altered by PTM. Therefore, PTM may be highly correlated with drug binding and PPI.

**Table 1:** Number of PTM proteins associated with PPI and drug binding

	All proteins	Human proteins
Total proteins	550,299	20,186
PTM proteins	112,985	13,080
PPI annotation (string)	326,227	17,833
PTM & PPI proteins	65,907	12,541
Drug-binding proteins(DrugBank)	–	1980
PTM & drug-binding proteins	–	1404

Indeed, an increasing number of studies are uncovering evidence of PTMs regulating drug-target interactions. For example, the epigenetic regulation of the chaperone cycle in different cell types or environmental conditions is found to involve changes in Hsp90 (heat shock protein 90) function through PTM [21]. Moreover, it has been shown that the effect of Hsp90 inhibitors could be enhanced when enzymes that facilitate the PTM of Hsp90 were suppressed [22], lending support to PTM being a potential therapeutic strategy for modulating the activity of Hsp90 in cancer cells. Phosphorylation, a common PTM of proteins, has also been utilized in drug-target design, whereby the interaction between the drug and the target is controlled by the state of phosphorylation [23, 24]. For instance, it is suggested that various upstream activators and different phosphorylation states can have a spectrum of effects on MEK inhibition, and therefore, greatly influence drug-target interaction with respect to MEK kinase pathway [25]. Since a large proportion of proteins undergo PTMs, it is likely that changes in PTMs regulate a drug’s efficacy and interaction with its target. Also, (Additional file 1: Table S1) shows that drugs can be categorized into two classes, small molecule drugs and biologics [26]. In general, most drugs are considered small organic compounds with a low molecular weight of less than 900 Da. Thus, for PTM studies in the context of drug-target binding, it would be reasonable to focus on the effects, frequency, and location of PTM near the site of binding by small molecule drugs.

The function of a protein can also be regulated by non-covalent PPIs [5, 27–31], a type of highly specific physical interactions between two or more protein molecules [32]. Many cellular processes are carried out through the complex interactions between various proteins, making up the interactome of a living cell or an organism. The binding affinities of these interacting proteins are also regulated by PTMs [5]. According to PTM data on the dbPTM database, more than 60% of PTM sites are found in the domains of proteins that actively participate in PPIs [18], providing support for a connection between PTM and PPI, and revealing the functions of the proteins involved in PPI. Thus, it is reasonable to assume that proteins capable of undergoing specific PTMs may exhibit certain properties related to PPI.

The diverse effects of PTMs on proteins, as well as their regulatory functions in various cellular processes contributed to the focus of this study—the investigation of drug-target binding and PPIs associated with PTM sites. In particular, we integrated protein tertiary structure and PPI information with the associated PTM sites from the annotations of 3did (3D interacting domains) [33]. PTM peptides were manually curated, and based on their sequence identity with records in the Protein Data Bank (PDB) [34] and UnitProtKB ID, mapped to their associated proteins. To uncover the impact on binding attributable to residues structurally surrounding PTM substrate, we investigated the orientations of side chains encompassing these neighboring residues in relation to the location of the PTM substrate sites in a protein structure. Finally, we constructed a database-assisted system, CruxPTM, to provide comprehensive information regarding PTM sites on protein tertiary structures, including the site-specific spatial composition of residues, surface area that is accessible to solvent, and residues that surround the PTM sites.

## Methods

**Fig. 1:**
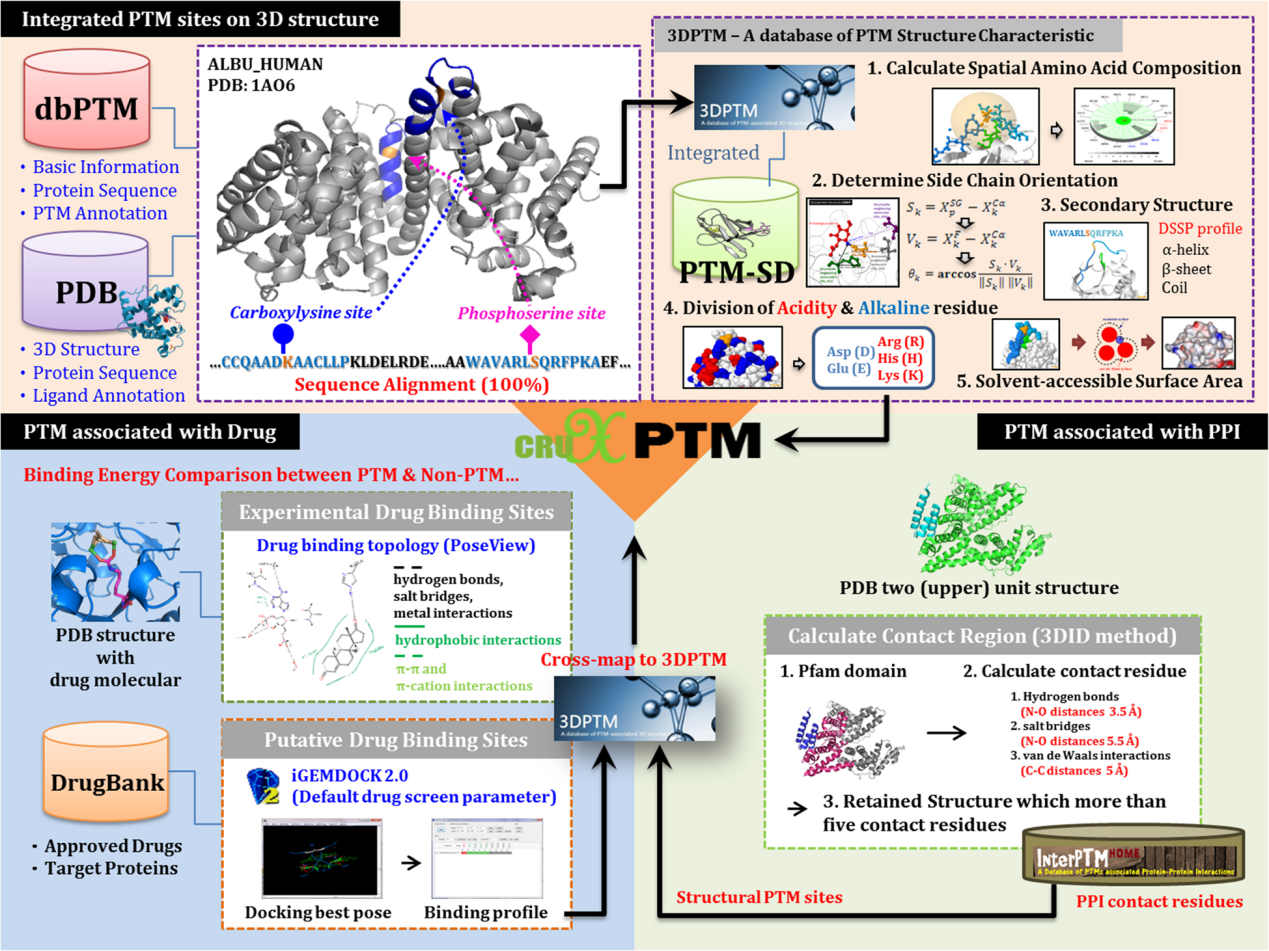
Flowchart of the analyses performed in this study. The experimentally verified PTM sites were acquired from dbPTM. Since drug binding sites and protein-protein interaction sites were extracted from protein structural information, we mapped all experimentally confirmed PTM sites to known 3D structures in the PDB by using UniProtKB ID and sequence identity. Then, the PTM sites were cross-matched with drug binding sites and PPI contacting sites for the identification of PTM sites associated with drug binding and PPI. Finally, these data were integrated with a PTM structural analytical method and computing programs for building up a web-based system

Figure 1 presents the workflow of this study. PTM sites that have been experimentally validated were acquired from dbPTM 3.0 [7], which is a useful database that comprehensively integrates all currently available PTM information. Since drug binding sites and protein-protein interaction sites were extracted from protein structural information, we mapped all experimentally confirmed PTM sites to known 3D structures from the PDB for subsequent analyses. Next, the PTM sites were cross-matched with drug binding sites and PPI contacting sites for the identification of PTM sites associated with drug binding and PPI. Finally, these data were combined with a PTM structural analytical method and computing programs for building up an online analysis platform. Detailed methods are as follows.

### Mapping of PTM sites to the tertiary structures of proteins

To identify the spatial composition of PTM substrate sites within the tertiary structures of proteins, we obtained from the PDB protein structures that have been determined by NMR or X-ray crystallography with an experimental resolution less than 2.5 Å [24]. According to the annotations in UniProtKB, 23,605 proteins in the PDB have 3D structure information. Also, chemical groups that can be covalently attached to the side chain of target residues were observed only in a few protein structures. Thus, to locate PTM substrate sites in 3D, mapping was performed between experimentally verified PTM peptides and the PDB protein records, and cross-referenced with the annotations of tertiary structures on UniProtKB with 100% similarity in sequence identity. Additionally, PTM sites possessing protein structures with modified residues were obtained from annotations on the PTM-SD database [19]. Most PTM sites that are mapped to structural sites are presented in the unmodified state, but PTM-SD provides complete information for modified PTM sites in 3D structures.

### Investigation of PTM sites associated with drugs binding

While it is suggested that the binding affinity of a small molecule can be regulated by a phosphorylation site within 12 Å of the site of binding [24], there is still a lack of information regarding the occurrences and influence of PTMs near drug-target binding. Therefore, we proposed a method in this study to identify PTM sites involved in drug binding. Figure 1 illustrates the workflow for extracting sites of drug-target binding in protein 3D structure. The entire process can be divided into two steps: 1) the processing of experimentally verified binding sites, and 2) molecular docking of drug binding. In step 1, we collected the structural information of small molecules that have associated keywords such as “drug,” “inhibitor,” “agonist” or “antagonist” and have drug annotations in the DrugBank [35]. A total of 34,555 PDB structures and 4803 small drug molecules which have DrugBank annotations were obtained. Then, the PoseView [36] method was employed to check the binding sites of each drug in the target proteins. PoseView provides a two-dimensional (2D) diagram showing how the drug ligand and the amino acid residues of the target protein may be arranged at the site of interaction. The nature of the interaction is presented in three ways. Black dashed lines indicate hydrogen bonds, salt bridges, and metal interactions. Green solid lines show hydrophobic interactions and green dashed lines represent π-π and π-cation interactions.

In step 2, a docking program, iGEMDOCK 2.0 [37], was utilized for the computational extraction of drug binding sites. We followed the four sequential steps in iGEMDOCK to perform the drug-target interaction analysis: target and database preparations, molecular docking and post-docking analyses. First, coordinates of the target protein atoms acquired from PDB, the ligand binding area, the atom’s formal charge and the atom types were specified. This procedure allowed iGEMDOCK to read the atom coordinates of a ligand from the prepared ligand database. After the ligand database and the target proteins were determined, docking was analyzed for each ligand using the flexible docking function provided by iGEMDOCK. The final step constituted the re-ranking and sorting of all docked ligand conformations based on an empirical scoring function and an evolutionary approach. The output of the program consisted of details regarding the docking result of each binding site, as well as the atomic characteristics of the target residues that interact with a specific drug ligand by hydrogen bonding (H), electrostatic (E) and van der Waal contact (V). A total of 1991 approved drugs from the DrugBank with 1632 target proteins were investigated by this proposed method. After mapping the experimentally verified PTM sites to the PDB structures, the PTM sites located in a drug binding site were determined to have strong associations with drug-binding, while those in the side chains that were within 12 Å of a drug-binding site were considered to be have relatively weak association with drug binding.

### Identification of PTM sites related to protein-protein interactions

In this work, the information of protein functional domains and PPIs were integrated for the identification of PTM-dependent protein interactions. To investigate the preferred functional domains of PTMs, we extracted the domain annotations from the Pfam database, which gives protein “signatures” based on protein families, domains and functional sites. In order to comprehensively study the structural properties of PTM sites associated with protein-interaction domains, the 3D structures of PPIs were acquired from the PDB. By adopting the 3D Interacting Domains (3DID) method proposed by Mosca et al. [33], the interaction interface of domain-domain interactions in the PDB 3D structures were determined as illustrated in Fig. 1. First we searched for protein structures with more than two subunits, and calculated the number of contact residues on the interface of the Pfam domain region containing the two subunits. Next, we applied a method based on previously published literature by Aloy and Russell [38], in which they derived the main-chain to side-chain and side-chain to side-chain potentials from the type of complexes described above. In particular, Aloy and Russell [38] defined interacting residues by using one or more of the following properties: hydrogen bonds (N-O distance of 3.5 Å), salt bridges (N-O distance of 5.5 Å), or van de Waals interactions (C-C distance of 5 Å). If there exists more than five pairs of residue contacts between two domains of a two-subunit region, these two subunits would be defined as an interaction structure. The contact residues were also extracted. A total of 30,455 PDB structures and 13,645 proteins were analyzed and 15,124 protein-protein interaction pairs were defined. Mapping between the experimentally verified PTM sites and the PDB structure uncovered PTM sites located on the PPI interfaces. These sites were regarded as PTM-driven PPIs.

## Results and discussion

### PTM substrate site characterization

**Fig. 2:**
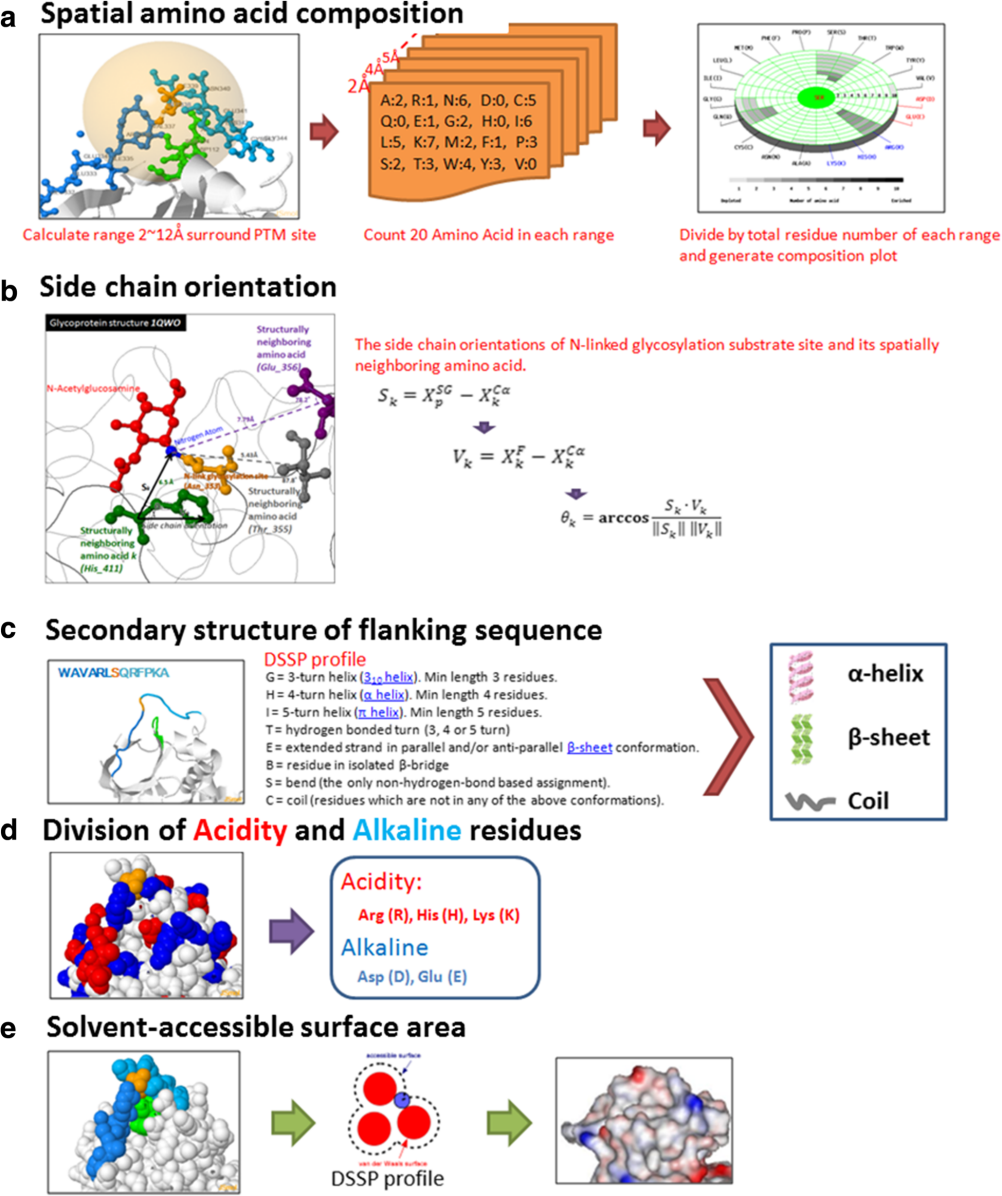
Investigation of the five structural characteristics for PTM substrate sites. To characterize PTM substrate sites, the structural characteristics such as (**a**) spatial amino acid composition, (**b**) the orientation of side chains around PTM substrate sites, (**c**) secondary structure of flanking sequences, (**d**) division of acidity and alkaline residues, and (**e**) solvent-accessible surface area were investigated

The availability of high-throughput proteomic technology has stimulated interests in understanding the structural environment of PTM substrate sites [17, 39]. To characterize PTM substrate sites, we considered a five-step approach, focusing on protein properties such as spatial amino acid composition, structurally neighboring residues and side chain orientations surrounding the PTM substrate sites, as well as the secondary structure, division of acidity and alkaline residues, and solvent-accessible surface area. In particular, we adopted the dictionary of protein secondary structure (DSSP) [40] for the calculation of solvent-accessible surface areas of proteins and for the standardization of PDB secondary structures with the corresponding PTM sites. To overcome difficulties that may arise during the derivation of substrate motifs from linear sequences [41], a radial cumulative propensity plot [42] was used to display the spatial composition and abundance of amino acids within and surrounding a particular PTM site (Fig. 2a). After a comprehensive and systematic analysis on the PDB structures, the number of PTM sites that can be mapped on protein structures is presented in (Additional file 2: Table S2). The spatial amino acid compositions were obtained by computing the relative frequencies of the 20 amino acids within 2 to 12 Å radial distances of the modified residues. Next, using JSmol software [43], neighboring amino acids at the sequence level and in the spatial context were presented with different colors on the PDB 3D structures for the structural characterization of PTM substrate sites. Following the method of Ruzza et al., the functional roles and drug binding effects associated with a PTM substrate site’s spatially neighboring residues were determined on the basis of these amino acids’ side chain orientations. As shown in Fig. 2b, given an N-linked glycosylation substrate site and its spatially neighboring amino acid, the vector from the residue to the nitrogen of N-linked glycosylated asparagine (*p*) is:

1 $$ {S}_k={X}_p^{SG}-{X}_k^{C\alpha} $$

where the crystallographic positions of the nitrogen in glycosylated asparagine *p* and the *C*_*α*_ atom in residue *k* are represented by ${X}_p^{SG}$ p and ${X}_k^{C\alpha}$, respectively. The vector *V*_*k*_ defines the direction of the side chain of a spatially neighboring amino acid *k* from its *C*_*α*_ atom to the functional atom (58):

2 $$ {V}_k={X}_k^F-{X}_k^{C\alpha} $$

where ${X}_k^F$ is the crystallographic positions of the functional atom, while ${X}_k^{C\alpha}$ is the *C*_*α*_ atom in residue *k*. The effect of the side chain of a spatially neighboring amino acid, *k*, on the substrate asparagine residue is defined by the angle *θ*_*k*_ between vectors *S*_*k*_ and *V*_*k*_:

3 $$ {\theta}_k=\mathbf{\operatorname{arccos}}\frac{S_k\bullet {V}_k}{\left\Vert {S}_k\right\Vert \left\Vert {V}_k\right\Vert } $$

The angle *θ*_*k*_ has to be less than 80° for the spatially neighboring amino acid *k* to be considered a functional residue to the asparagine on the N-linked glycosylation [17, 44].

### Case study of PTM sites associated with drug binding

**Fig. 3:**
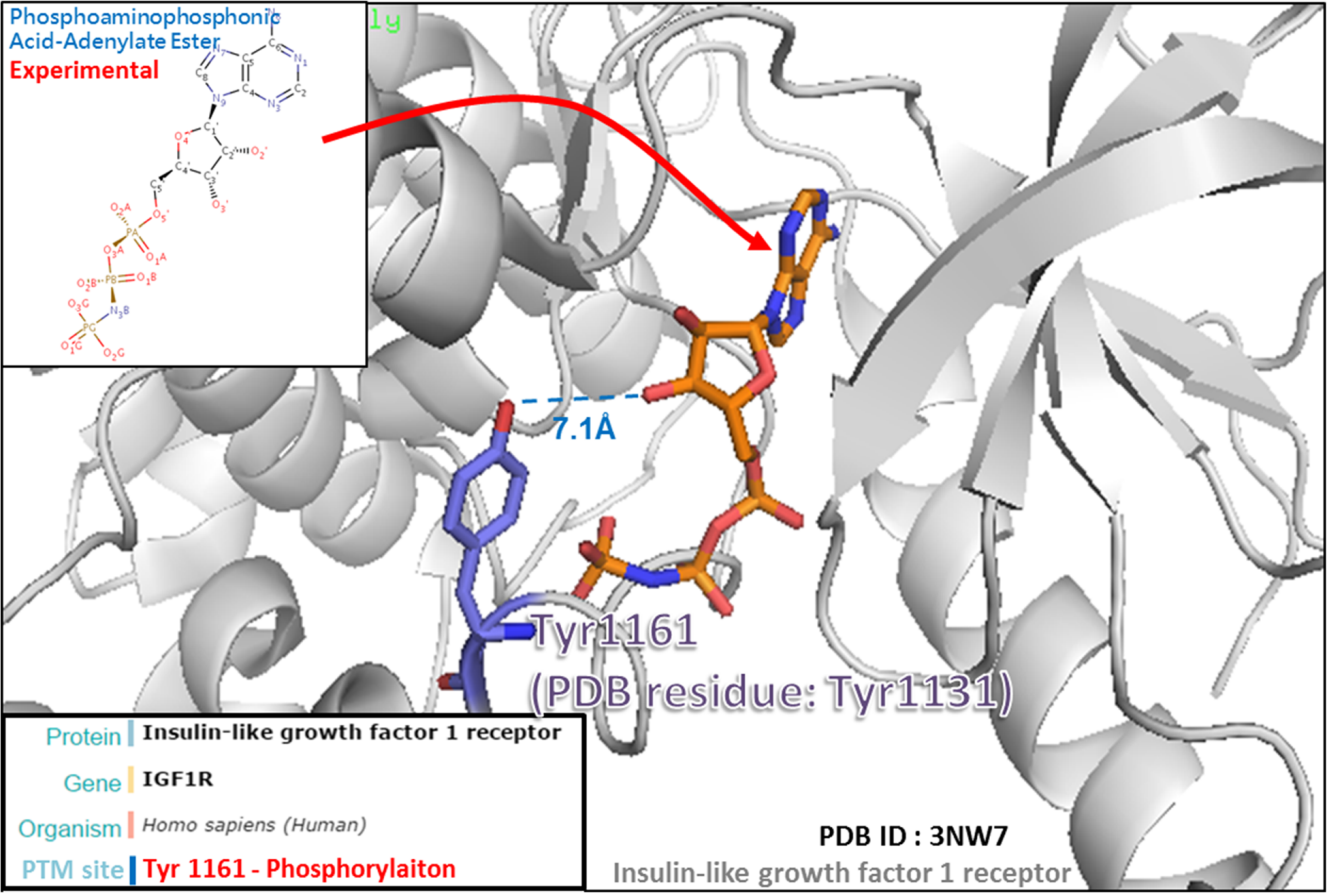
A case study of the Tyr1131 phosphorylation site associated with drug binding on insulin-like growth factor 1 receptor (IGF1R). The IGF1R is a type of kinases and an inhibitor of the IGF1R could maintain the protein in an inactive conformation. Since the IGF1R was phosphorylated, the crystal structure of its activation loops would be rearranged in such a way that significantly decreases the inhibitor’s affinity. Thus, Tyr1131 phosphorylation site may provide a functional role by modulating the structure of the target protein and reducing the affinity between the inhibitor and the target site

More than 1100 PTM substrate sites related to drug binding were curated and archived in CruxPTM after a large-scale screening for PTM substrate sites and drug-binding sites in the PDB. The number of drug binding associated sites for each PTM type can be found in Table 2. Most of the drug binding sites appeared to be able to undergo phosphorylation, while the second most common PTM among the drug binding sites seemed to be ubiquitylation. According to dbPTM [6], the phosphorylation state of Ser843, situated close to the drug binding site (6.4 Å), could influence the affinity of binding for the agonist and inhibitor of the mineralocorticoid receptor (MCR). This is supported by the observation that posphorylated Ser843 reduces the MCR’s binding affinity for its agonist and leads to the receptor’s own inactivation [45]. Phosphorylation does not always inhibit the protein’s activity. In the case of most kinases, while reducing the affinity between a drug and its target, phosphorylation can actually increase the activity of the target protein [11, 46–48]. The insulin-like growth factor 1 receptor (IGF-1R) is an example of the type of kinases (Fig. 3). A case study of IGF-1R shows that an inhibitor of the receptor could maintain the protein in an inactive conformation; however, if the receptor becomes phosphorylated, the crystal structure of its activation loops would be rearranged in such a way that significantly decreases the inhibitor’s affinity for the receptor while enhancing the activity of the receptor. Therefore, phosphorylation may affect the efficacy of a drug by modulating the structure of the target protein and reducing the affinity between the drug and the target.

**Table 2:** Number of PTM sites associated with drug binding sites

PTM Instances	Number of PTM sites located on drug contact sites	Number of PTM sites within 12 Å distance of drug contact sites
Phosphorylation	400	1032
Ubiquitylation	164	331
Acetylation	113	296
Pyridoxal phosphate	84	103
N-linked Glycosylation	82	375
O-linked Glycosylation	51	50
S-nitrosylation	38	47
Methylation	25	92
Disulfide bond	18	34
FAD	17	0
Gamma-carboxyglutamic acid	17	32
Proteolytic Cleavage	17	68
C-linked Glycosylation	15	23
N6-carboxylysine	15	12
Carboxylation	12	30
Nucleotide-binding	8	5
Glutathionylation	6	0
Myristoylation	6	10
N6-succinyllysine	6	21
Palmitoylation	6	26
Dephosphorylation	5	5
Oxidation	5	12
FMN	4	0
Prenylation	4	3
Thioether bond	4	19
TPQ	4	0
Deamidation	3	0
Dehydroxylation	3	0
Glycation	3	0
Isopeptide bond	3	0
Neddylation	3	0
Pyruvate	3	0
Sumoylation	3	47
Tryptophylquinone	3	0
ADP-ribosylation	2	3
Biotin	2	0
Deacetylation	2	2
Hydroxylation	2	13
Lipoprotein	2	0
Nitration	2	10
Phosphopantetheine	2	0
Pyrrolidone carboxylic acid	2	21
Tryptophyl-tyrosyl-methioninium	2	0
Allysine	1	0
Amidation	1	36
Carbamidation	1	0
Chromophore	1	0
Formylation	1	1
Lipoyl	1	0
N6-malonyllysine	1	0
S-linked Glycosylation	1	0
Sulfation	1	3
Transglutamination	1	0
TTQ	1	0
Total	1189	2762

**Fig. 4:**
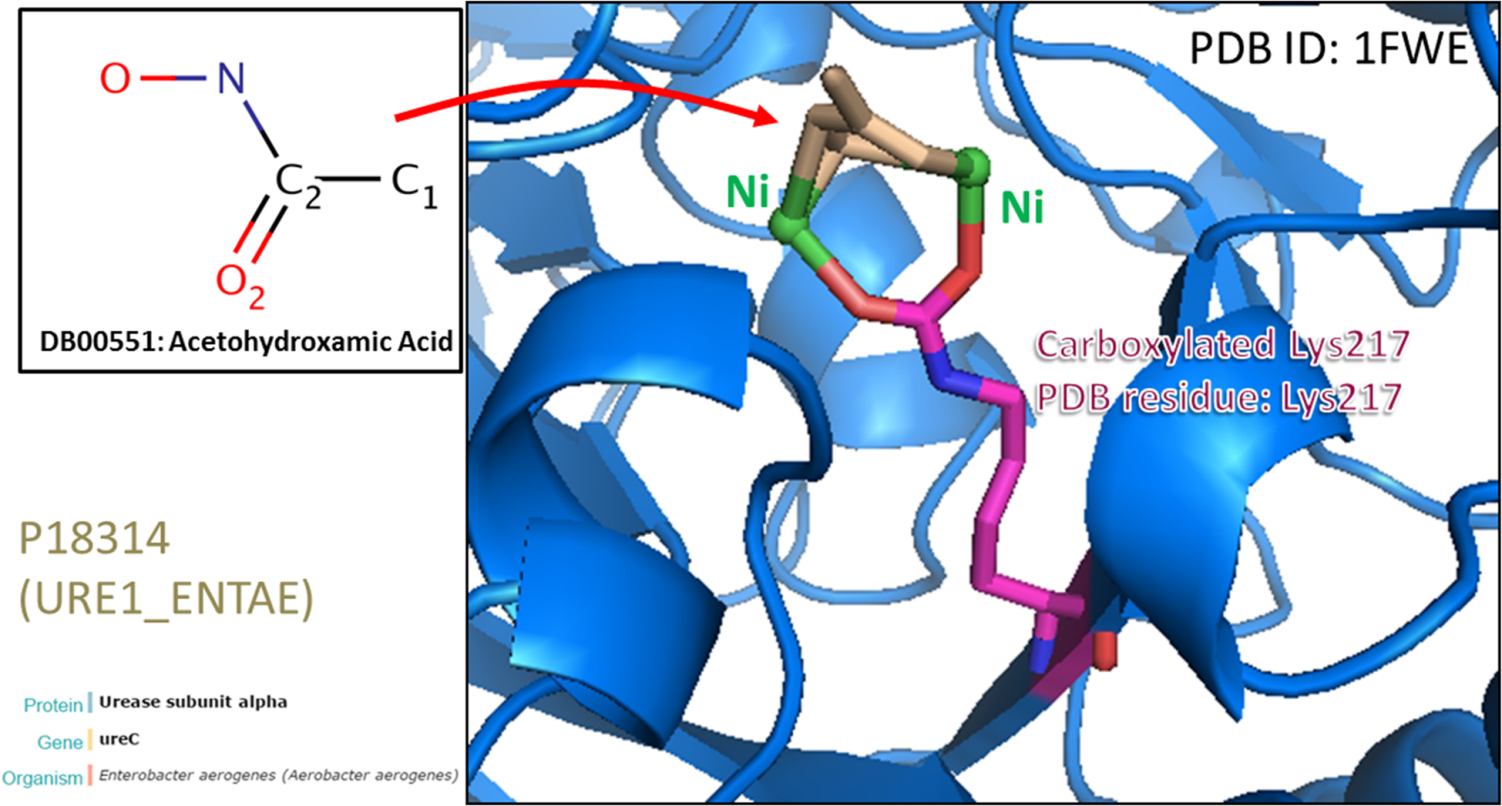
A case study of the Lys217 carboxylation site associated with drug binding on urease subunit alpha (URE1). Urease is responsible for hydrolyzing urea into carbon dioxide and ammonia. The active site of all known ureases is composed of a bis-μ-hydroxo dimeric nickel center, located in the alpha (α)-subunit, and has an interatomic distance of ~3.5 Å. Our analysis shows that acetohydroxamic acid might inhibit urease activity by competing with nickel atoms in the enzyme to form a chelate. This could potentially interrupt the hydrolysis (Lys217 carboxylation) of urea, which reduces the concentration of urinary ammonia and lowers urine pH

With reference to a case study discussed on dbPTM, an acetylation site (Lys199) on human serum albumin (HSA) is essential for drug transport and metabolism [49]. Annotations on the OMIM database [50] implicates HSA in hyperthyroxinemia (OMIM ID: 615,999) and analbuminemia (OMIM ID: 616,000). Accordingly, this investigation shows that the Lys199 residue is 4.3 Å from the salicylic acid (DrugBank ID: DB00936) binding site. Aspirin (DrugBank ID: DB00945) has been found to be able to acetylate Lys199, while being hydrolyzed into salicylic acid by HAS [6]. Thus, our investigation confirmed the conformational plasticity of HSA and provided a possible explanation for the regulation of HSA-drug interaction through PTM at the Lys199 residue. Figure 4 shows another example of the modulation of drug-target interaction through PTM. Urease is responsible for hydrolyzing urea into carbon dioxide and ammonia. Carbaoxylation of the Lys217 residue on the *Klebsiella aerogenes* urease coordinates the contact between two nickel ions and the drug molecule acetohydroxamic Acid (DrugBank ID: DB00551). The active site of all known ureases is composed of a bis-μ-hydroxo dimeric nickel center, located in the alpha (α)-subunit, and has an interatomic distance of ~3.5 Å [51]. Our analysis shows that acetohydroxamic acid might inhibit urease activity by competing with nickel atoms in the enzyme to form a chelate. This could potentially interrupt the hydrolysis of urea, which reduces the concentration of urinary ammonia and lowers urine pH.

### Functions of PTM sites on protein-protein interactions

As shown in (Additional file 3: Table S3), of all the experimentally verified PTM sites, over 20% can be found in the functional domains of proteins, implicating the biological significance of PTMs. We studied these sites to infer the roles that these PTMs play in PPI interactions. For instance, approximately 70% of known *S*-nitrosylation sites, which are responsible for the regulation of NO-related cellular processes, are located in functional domains. Also, among the data that we have collected for the current study, more than 1900 PTM sites are localized to the interface of domain-domain interacting regions. Based on our observations, it appears that structural associations exist between many PTM sites and binding sites for specific PPI domains and perhaps even regulate the interactions between proteins by modifying the sites of contact.

**Fig. 5:**
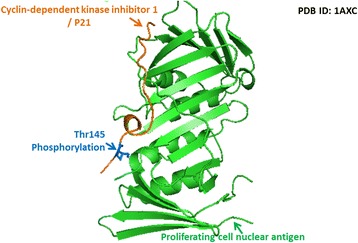
A case study of the Thr145 phosphorylation site located in the interacting region of p21–PCNA complex (PDBID: 1AXC). The phosphorylation of the Thr145 residue of p21, which corresponds to the PCNA binding region (from residue 144 to 151), may inhibit the interaction between p21 and PCNA, resulting in PCNA binding with other DNA polymerase components [54]

Cyclin-dependent kinase inhibitor p21, by binding to cyclin-dependent kinases (CDKs), acts as an important checkpoint in cell cycle arrest in response to DNA damage [52]. It can also bind to proliferating cell nuclear antigen (PCNA) to suppress DNA replication [53]. While in solution, the p21 protein does not exhibit a stable structure. However, upon binding to target proteins, the protein assumes an ordered stable conformation. Figure 5 shows that phosphorylation of the Thr145 residue of p21, which corresponds to the PCNA binding region (from residue 144 to 151), may inhibit the interaction between p21 and PCNA, resulting in PCNA binding with other DNA polymerase components [54].

**Fig. 6:**
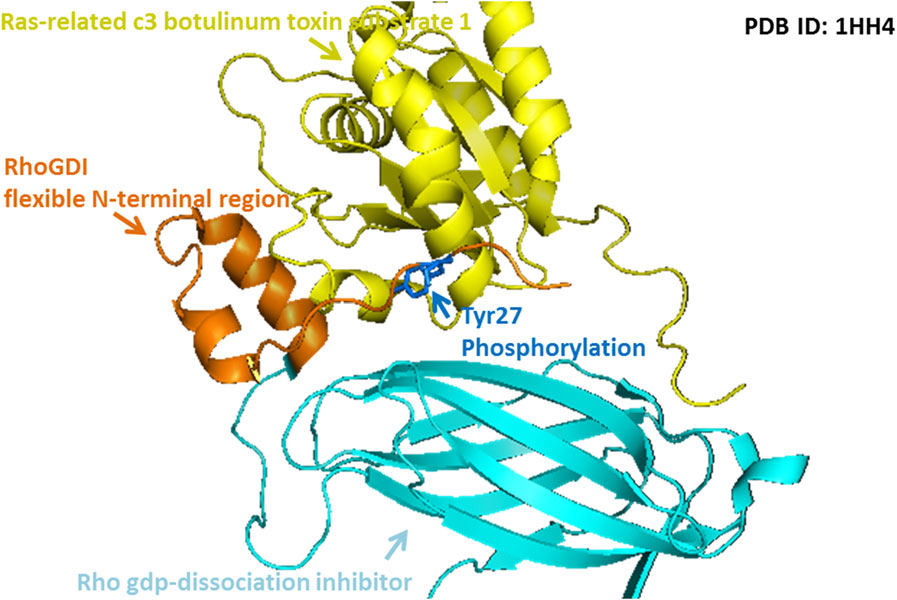
A case study of the Tyr127 phosphorylation site located in the interacting region of RhoGDI–Rac1 complex (PDBID: 1HH4). A disordered N-terminal domain of RhoGDI1 contains a tyrosine residue (Tyr27), which is localized to the docking interface. The phosphorylated Tyr127 has been reported to facilitate the dissociation of RhoA, Rac1, and cdc from RhoGDI1, making GTPases available for activation [57]

The Rho GDP-dissociation inhibitor 1 (RhoGDI1) is a regulator of the Rho family GTPase [55]. By preventing the release of GDP and the loading of GTP on Rho proteins, RhoGDIs can inhibit the activity of Rho family GTPase. RhoGDIs can also block the degradation or improper activation of inactive Rho proteins by transferring them to the cell membrane. The function of RhoGDIs is regulated by phosphorylation at their Ser, Thr and Tyr residues. In fact, phosphorylation at multiple sites in RhoGDIs can stimulate the simultaneous release of multiple Rho proteins [55]. The key functional region of RhoGDIs lies in its N-terminal domain. This region, though generally disordered, can form two helices and bind to the switch I and switch II regions of GTPase to prohibit the latter from making conformational changes required for the exchange of GDP and GTP [56]. As presented in Fig. 6, the disordered N-terminal domain of RhoGDI1 contains a Tyr residue (Tyr27), which is localized to the binding interface and can be phosphorylated to facilitate the dissociation of RhoA, Rac1, and cdc from RhoGDI1, making GTPases available for activation [57].

**Fig. 7:**
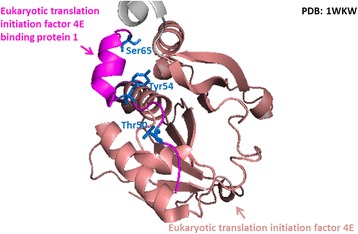
A case study of the phosphorylation sites located in the interacting region of the ternary complex of eIF4E-m7GpppA-4EBP1 peptide (PDBID: 1WKW). There are three substrate sites (Thr50, Tyr54 and Ser65) of phosphorylation within the binding region of the 4EBP1, which can regulate its interaction with eIF4E. These sites are reported to modulate the reversible binding of 4EBP1 with eIF4E, and hyper-phosphorylation at these sites decreases the strength of interaction between the two proteins [59]

The crystal structure of the ternary complex of the eIF4E-m7GpppA-4EBP1 peptide is shown in Fig. 7. This structure, formed from the interaction among the 7-methylguanosine at the 5′-cap of mature transcripts, eukaryotic initiation factor 4E (eIF4E), and endogenous 4E–binding protein 1 (4EBP1), is required for translation initiation [58]. Figure 7 indicates that three substrate sites (Thr50, Tyr54 and Ser65) of phosphorylation are located within the binding region of the 4EBP1, which can regulate its interaction with eIF4E. The three sites are reported to modulate the reversible binding of 4EBP1 with eIF4E, and hyper-phosphorylation at these sites can decrease the strength of interaction between the two proteins [59].

## Conclusion

In this study, we first mapped PTM sites to the 3D structures of proteins, and adopted multiple methods to describe the structural characteristics of PTM sites in tertiary structures. Already, studies are emerging that use similar methods to investigate PTM; for example, Karabulut and Frishman’s study [60] that utilizes spatial amino acid composition to identify acetylation sites. However, by employing several different approaches and considering several structural characteristics of a variety of PTM sites associated with drug-target binding and PPI, this work can effectively facilitate the functional study of various types of PTM. Indeed, the reliability of our analysis can be supported by the fact that other studies also identified some of the drug-binding and PPI associated PTM sites uncovered in our investigation.

Our approach has the potential to be applied on drug design, which often centers around the influence of amino acid mutation on the effect of a drug. However, PTMs are also affected by changes in the amino acid sequence. Our study indicates that PTMs can be crucial to a drug’s effect on a structural level, and knowing PTM sites associated with protein-protein interaction is helpful for understanding the biological mechanisms involving these PTM sites.

For situations where information regarding the protein’s structure is lacking, we attempted to overcome this limitation with molecular docking. According to the latest statistics from the PDB in 2016, there are over 122,000 records for protein structures. Although the number of annotated PDB structures is increasing rapidly, information of structural proteins is still limited. When cross-referenced with annotations on UniProtKB, it was found that only 23,605 out of 551,705 reviewed proteins and 12,165 out of 114,895 PTM proteins have crystal structure information, respectively. Some proteins only have partially annotated crystal structure related to specific fragments in their sequences such that it was impossible to map the PTM sites to these proteins’ 3D structures. For example, the ankyrin-3 protein have 16 experimental and 17 putative PTM sites within its sequence of 4377 amino acids, but only the region between amino acid 4088 and 4199 has annotated crystal structure. As a result, only one PTM site could be mapped to this structure. This limitation may affect the reliability of comparison among PTM sites.

CruxTPM is a novel, integrative web platform for the analysis of PTMs and their biological roles in a 3D structural context. It enables the structural characterization and 3D visualization of PTM sites, as well as the investigation of their relationship with drug-target binding and PPI. The tool also provides interactive function like drug structure search, PTM modified structure visualization, online small molecule docking, etc. We hope this study and analytical platform can help enhance the understanding of the biological mechanisms associated with PTMs and improve the efficiency of drug design.

## Additional files

Additional file 1: Table S1. Classification and definition of drugs. (PDF 70 kb)

Additional file 2: Table S2. Number of PTM sites that can be mapped to the PDB 3D structures. (PDF 41 kb)

Additional file 3: Table S3. Number of PTM sites located in protein-protein interaction regions. (PDF 155 kb)
