# Supplementary material for: Investigation and identification of functional post-translational modification sites associated with drug binding and protein-protein interactions
Source: BMC Syst Biol. 2017 Dec 21;11(Suppl 7):132. doi: 10.1186/s12918-017-0506-1 (PMC5763307; doi:10.1186/s12918-017-0506-1)
Supplement: Supplementary file 1 — Classification and definition of drugs. (PDF 70 kb) [file 12918_2017_506_MOESM1_ESM.pdf]

Table S1. Classification and definition of drugs.

	Small molecules	Biologics (Big molecules)	
		**Small biologics**	**Biologics**
**Atomic size**	20-100 atoms	200-3000 atoms	500-50,000 atoms
**Molecular weight**	< 900 Dalton	Thousand to hundred thousand Dalton	
**Example**	Aspirin	Hormones, Cytokines	EPO, Avastin
**Usage**	Multiple, Mainly oral	Injection	
**Production**	Natural extraction & Synthetic	Produced by recombinant DNA or Cell culture (E.coli, Yeast, Mammal cell…)	
