# Supplementary material for: Investigation and identification of functional post-translational modification sites associated with drug binding and protein-protein interactions
Source: BMC Syst Biol. 2017 Dec 21;11(Suppl 7):132. doi: 10.1186/s12918-017-0506-1 (PMC5763307; doi:10.1186/s12918-017-0506-1)
Supplement: Supplementary file 2 — Number of PTM sites that can be mapped to the PDB 3D structures. (PDF 41 kb) [file 12918_2017_506_MOESM2_ESM.pdf]

Table S2. Number of PTM sites that can be mapped to the PDB 3D structures.

PTM type	Number of substrate sites mapped on PDB 3D structures	Number of PTM site annotated on PDB 3D structures
N-linked Glycosylation	2098	1090
Phosphorylation	11084	287
Gamma-carboxyglutamic acid	134	88
O-linked Glycosylation	209	82
Pyrrolidone carboxylic acid	77	67
Hydroxylation	81	59
Methylation	358	29
Acetylation	2717	24
N6-carboxylysine	27	22
Sulfation	27	11
Oxidation	37	7
Formylation	15	6
S-nitrosylation	613	5
Pyridoxal phosphate	110	3
Bromination	2	1
Lipoyl	6	1
Nitration	25	1
Pyruvate	7	1
Retinal protein	8	1
Ubiquitylation	6144	0
Disulfide bond	717	0
Proteolytic Cleavage	308	0
Sumoylation	176	0
Amidation	109	0
N6-succinyllysine	79	0
Isopeptide bond	70	0
Dephosphorylation	65	0
Thioether bond	53	0
Carboxylation	43	0
Palmitoylation	38	0

**C-linked Glycosylation**	38	0
**Neddylation**	37	0
**Caspase**	33	0
**Myristoylation**	26	0
**Dehydroxylation**	25	0
**Deamidation**	20	0
**N6-malonyllysine**	19	0
**FAD**	17	0
**Cyclopeptide**	17	0
**Prenylation**	14	0
**Nucleotide-binding**	13	0
**Glutathionylation**	12	0
**ADP-ribosylation**	12	0
**TPQ**	9	0
**GPI-anchor**	8	0
**Citrullination**	8	0
**Tryptophyl-tyrosyl-methioninium**	6	0
**Tryptophylquinone**	5	0
**Thioester bond**	5	0
**Phosphopantetheine**	5	0
**Lipoprotein**	5	0
**FMN**	5	0
**D-amino acid and Thioether bond**	5	0
**Allysine**	5	0
**Alkylation**	5	0
**Transglutamination**	4	0
**Hypusine**	4	0
**Deacetylation**	4	0
**D-amino acid**	4	0
**TTQ**	3	0
**Biotin**	3	0
**S-linked Glycosylation**	2	0
**Organic radical**	2	0

**Methylation and Thioether bond**	2	0
**LTQ**	2	0
**Diphthamide**	2	0
**Deglycosylation**	2	0
**CTQ**	2	0
**Carbamidation**	2	0
**Selenocysteine**	1	0
**N6-1-carboxyethyl lysine**	1	0
**Lysinoalanine**	1	0
**Glycyl Serine isopeptide**	1	0
**Covalent protein-DNA linkage and Phosphorylation**	1	0
**Chromophore**	1	0
**total**	25835	1785
