# Supplementary material for: Investigation and identification of functional post-translational modification sites associated with drug binding and protein-protein interactions
Source: BMC Syst Biol. 2017 Dec 21;11(Suppl 7):132. doi: 10.1186/s12918-017-0506-1 (PMC5763307; doi:10.1186/s12918-017-0506-1)
Supplement: Supplementary file 3 — Number of PTM sites located in protein-protein interaction regions. (PDF 155 kb) [file 12918_2017_506_MOESM3_ESM.pdf]

Table S3. Number of PTM sites located in protein-protein interaction regions.

PTM Type	Number of PTM sites in PPI interface	Number of PTM sites in functional domain
**Phosphoserine**	296	992
**N-linked (GlcNAc...)**	411	1472
**Phosphothreonine**	161	561
**N6-acetyllysine**	238	557
**Phosphotyrosine**	110	275
**N-acetylalanine**	2	4
**O-linked (GalNAc...)**	42	117
**N-acetylserine**	4	12
**N6-(pyridoxal phosphate)lysine**	139	290
**4-carboxyglutamate**	0	1
**Pyrrolidone carboxylic acid**	10	24
**N-acetylmethionine**	0	5
**N-linked (Glc) (glycation)**	193	347
**S-palmitoyl cysteine**	0	9
**N6-succinyllysine**	36	47
**N6-methyllysine**	2	15
**4-hydroxyproline**	1	3
**N-myristoyl glycine**	0	4
**N6-carboxylysine**	0	26
**Sulfotyrosine**	8	26
**S-nitrosocysteine**	49	78
**Blocked amino end**	0	2
**(3S)-3-hydroxyasparagine**	5	7
**Deamidated asparagine**	17	23
**N6,N6,N6-trimethyllysine**	10	12
**O-linked (Xyl...)**	3	5
**N-acetylthreonine**	0	1
**S-geranylgeranyl cysteine**	0	1
**O-linked (GlcNAc)**	0	1
**C-linked (Man)**	0	0

**S-farnesyl cysteine**	0	0
**5-hydroxylysine**	0	0
**Glutamate methyl ester**	1	3
**Cysteine methyl ester**	0	1
**O-linked (Gal...)**	0	0
**N-formylmethionine**	0	3
**N-palmitoyl cysteine**	0	1
**Phosphohistidine**	9	14
**Hydroxyproline**	0	0
**N4-methylasparagine**	0	10
**4-aspartylphosphate**	0	8
**Asymmetric dimethylarginine**	0	0
**2',4',5'-topaquinone**	1	7
**Pyruvic acid (Ser)**	6	7
**N6,N6-dimethyllysine**	0	6
**N-acetylglycine**	0	0
**S-8alpha-FAD cysteine**	0	6
**S-diacylglycerol cysteine**	15	21
**O-AMP-tyrosine**	25	36
**Omega-N-methylated arginine**	0	0
**O-linked (Fuc...)**	1	1
**N6-lipoyllysine**	4	12
**Cysteine sulfinic acid (-SO2H)**	14	26
**N-acetylvaline**	0	0
**ADP-ribosylarginine**	2	4
**N-acetylproline**	0	1
**Cysteine sulfenic acid (-SOH)**	0	5
**Nitrated tyrosine**	8	10
**GPI-anchor amidated serine**	0	0
**N-acetylaspartate**	0	3
**ADP-ribosylcysteine**	0	2
**(3R)-3-hydroxyaspartate**	4	9
**O-linked (Fuc)**	21	33

**N6-crotonyl-L-lysine**	0	2
**N6-myristoyl lysine**	0	0
**3-hydroxytryptophan**	0	4
**S-methylcysteine**	0	2
**S-cysteinyl cysteine**	0	1
**O-(pantetheine 4'-phosphoryl)serine**	0	2
**Tele-methylhistidine**	4	13
**Methionine sulfoxide**	6	10
**N6-(retinylidene)lysine**	0	3
**Omega-N-methylarginine**	0	1
**(3R)-3-hydroxyasparagine**	1	1
**O-AMP-threonine**	26	33
**Tele-8alpha-FAD histidine**	0	3
**Citrulline**	1	1
**Tryptophylquinone**	0	3
**N6-malonyllysine**	0	2
**(Z)-2,3-didehydrotyrosine**	1	3
**Deamidated glutamine**	0	2
**Pros-methylhistidine**	0	3
**(E)-2,3-didehydrotyrosine**	5	7
**S-4a-FMN cysteine**	0	2
**N-methylmethionine**	0	0
**Dimethylated arginine**	0	0
**O-linked (Glc...)**	4	6
**2,3-didehydroalanine**	4	4
**Lysine amide**	0	0
**5-methylarginine**	0	0
**1-thioglycine**	0	0
**2-(S-cysteinyl)pyruvic acid O-phosphothioketal**	0	2
**Glycine amide**	0	0
**O-linked (Man)**	0	0
**Cysteine persulfide**	0	2

**N-methylalanine**	0	0
**3-methylthioaspartic acid**	0	2
**Methionine amide**	1	1
**3-hydroxyhistidine**	0	0
**(3S)-3-hydroxyaspartate**	0	1
**Phosphatidylethanolamine amidated glycine**	0	0
**O-linked (GlcNAc...)**	0	0
**N2-acetylarginine**	0	0
**N-D-glucuronoyl glycine**	0	0
**O-8alpha-FAD tyrosine**	3	5
**Tele-(1,2,3-trihydroxypropan-2-yl)histidine**	0	1
**S-6-FMN cysteine**	0	1
**Lysine methyl ester**	0	0
**Diphthamide**	0	1
**Serine amide**	0	0
**N-methylphenylalanine**	9	11
**O-(sn-1-glycerophosphoryl)serine**	0	1
**O-linked (DADDGlc...)**	0	1
**Phenylalanine amide**	0	1
**Arginine amide**	0	0
**N-palmitoyl glycine**	0	0
**N-methylproline**	0	0
**N6-methylated lysine**	0	0
**N-pyruvate 2-iminyl-valine**	0	0
**S-(dipyrrolylmethanemethyl)cysteine**	0	1
**Cholesterol glycine ester**	0	0
**Hypusine**	0	1
**O-acetylthreonine**	0	0
**S-glutathionyl cysteine**	0	1
**N6-murein peptidoglycan lysine**	0	0
**2-methylglutamine**	0	1

**N-formylglycine**	0	1
**Glutamine amide**	0	1
**N-acetylcysteine**	0	0
**Cis-14-hydroxy-10,13-dioxo-7-heptadecenoic acid aspartate ester**	0	1
**N6-palmitoyl lysine**	2	3
**Leucine amide**	1	5
**O-linked (Man...)**	0	1
**Glycine radical**	0	1
**O-(5'-phospho-RNA)-tyrosine**	0	0
**S-stearoyl cysteine**	0	0
**Leucine methyl ester**	0	0
**N6-biotinyllysine**	1	1
**S-(4-hydroxycinnamyl)cysteine**	0	1
**O-(2-cholinephosphoryl)serine**	0	1
**Symmetric dimethylarginine**	0	1
**Methionine derivative**	0	0
**N5-methylglutamine**	0	1
**N,N,N-trimethylalanine**	0	0
**(3R)-3-hydroxyarginine**	0	1
**N,N-dimethylproline**	0	0
**Cysteine methyl disulfide**	0	1
**3',4'-dihydroxyphenylalanine**	0	1
**Total**	1917	5309
